# The Development of a Novel, Standards-Based Core Curriculum for Community Facing, Clinic-Based Community Health Workers

**DOI:** 10.3389/fpubh.2021.663492

**Published:** 2021-06-07

**Authors:** Sheba George, Lucero Silva, Myra Llamas, Irma Ramos, Justin Joe, Juan Mendez, Rosalva Salazar, Jim Tehan, Tatiana Vasquez, Schetema Nealy, Hector Balcazar

**Affiliations:** ^1^Department of Preventive and Social Medicine, College of Medicine, Charles R. Drew University of Medicine and Science, Los Angeles, CA, United States; ^2^Department of Community Health Sciences, University of California, Los Angeles, Los Angeles, CA, United States; ^3^College of Science and Health, Charles R. Drew University of Medicine and Science, Los Angeles, CA, United States; ^4^Providence Health Systems, Los Angeles, CA, United States

**Keywords:** community health workers, training for community health workers, standards-based curriculum for community health workers, community health workers in clinical settings, CHW Consensus Project (C3 Project)

## Abstract

**Introduction:** Historically, CHW trainings have been developed to support community-based CHWs. When CHWs have been trained to engage with patients, typically such trainings have been for short term grant funded projects, focusing on a specific health intervention and not for long term, ongoing engagement of CHWs employed in clinical settings. To the best of our knowledge, this is the first such effort to describe the development of a standards-based training curriculum for clinic-based CHWs using a novel conceptual framework.

**Methods:** Our conceptual approach for curricular development has several innovative features including: (1) a foundational consultation process with CHW national experts to inform curricular development approach, process and content; (2) utilization of the CHW Consensus Project (C3 Project) to provide curricular standards and guide learning objectives; (3) integration of three key stakeholder group perspectives (patients, healthcare teams, and healthcare systems); (4) use of popular education principles, aiming to foster a collaborative learning process; (5) integration of adult learning principles which build on learners' experiences, culminating in a modified apprenticeship model and (6) collaboration with clinical partners throughout planning and development of the curriculum.

**Results:** The resulting standards-based curriculum is comprised of 10 modules, which span three areas of focus: (1) Establishing a professional CHW identity and competencies; (2) Outlining the context, processes and key actors in health care settings with whom CHWs will engage; and (3) Identifying the main forces that shape health and health care outcomes of patients/families and communities.

**Discussion:** We highlight four lessons from our curriculum development process that may help other such efforts. First, curricular development should utilize CHW standards, existing training materials, and community-focused principles to inform curricular content and learning outcomes. Second, curricula should support training delivery using experience-based, participatory approaches, consistent with adult education and popular education principles. Third, training development for clinical settings should also draw from clinical CHW experiences and input. Fourth, curricula should support training for key stakeholders and champions in clinical organizations to improve organizational readiness for integrating CHWs into healthcare teams and health systems. Our results contribute to growing research on effective CHW training methods for clinical settings.

## Introduction: Background and Rationale for the Educational Activity Innovation

Community health workers (CHWs) can be integral components of clinical care teams, having a long history of functioning as frontline public health staff who conduct outreach and build trust with vulnerable populations in federally qualified health centers (FQHCs), hospitals, public health agencies, and through community-based organizations. They have played an increasingly important role in health interventions/programs, often bridging the gap between clinic and community by facilitating care coordination (1, 2), health promotion (3), and communication between clinicians and patients/program participants (4) in a manner that is generally assumed to be more acceptable to the care recipients and ultimately improving health outcomes (5–7). CHW interventions have been identified as an essential strategy to address health disparities for patient-centered medical home (PCMH) (8–10) by the NHLBI (11) and the Centers for Disease Control and applauded for their contributions to the Institute for Healthcare Improvement's Triple Aim objectives (2, 12–14).

In healthcare settings, CHWs are intermediaries between patients and healthcare institutions and can help improve health outcomes. Because CHWs are often embedded in the community and are uniquely able to bridge the gap between healthcare organizations and the safety net patients in the community, CHWs are in a great position to address the potential obstacles to patient-centered care. With the onset of COVID-19, on March 19, 2020, the Department of Homeland Security's Cybersecurity and Infrastructure Security Agency issued a memorandum which included CHWs in the list of “essential critical infrastructure workers who are imperative during the response to the COVID-19 emergency for both public health and safety as well as community well-being” (15). Sen. Kirsten Gillibrand (D-NY) and Sen. Michael Bennet (D-CO) have proposed creating a national Health Force, inspired by the Depression-era Works Progress Administration, to recruit, train, and employ “hundreds of thousands” of CHWs to perform contact tracing and testing and provide a range of services (16). From much of the existing literature, including our own recent systematic review of patient feedback on CHWs' care provision (17), it is evident that CHWs are well-positioned to build trust surrounding clinical directives, provide credible health care information, and address barriers related to the social determinants of health. If CHWs are trained appropriately and integrated into clinical settings, such a patient-engagement and community outreach strategy addressing social determinants of health within a public health framework with the CHW at the center can provide a sustainable, new paradigm for meeting not only COVID-19 testing needs but also for engaging patients in accessing COVID-19 vaccinations and other emergent health care challenges.

Historically, CHW trainings have often been developed to support community-based CHWs. When CHWs have been trained to engage with patients, typically such trainings have historically been for short term grant funded projects, focusing on a specific health intervention and not geared toward a long term, ongoing engagement of CHWs employed in clinical settings but this is beginning to change. However, the lack of national consensus and the wide spectrum of CHW practice has contributed to a variety of CHW trainings that vary in scope of practice and are often limited to disease interventions or specific patient populations (18). The research on CHWs also tends to not address the topic of training with O'Brien et al. finding that only 41% of articles addressing this topic in their 2009 review (19). Despite their potential vitality to health care teams and their wide scope of practice, CHW training is not standardized. For example, there are no formal training or certification requirements for CHWs in California (20). Nationwide, the work of CHWs is just as varied since educational and training requirements of CHWs vary from state to state (21).

Part of the challenge is the disagreement about whether it is necessary to standardize CHW work and training. Standardizing CHW training with curriculum in academic settings raises concerns as to whether CHWs will be able to maintain their community identity that is so crucial to their practice and the effect it would have on the existing workforce (18, 22). Despite concerns about standardization, there have been several local and national efforts to identify standard roles and competencies for CHWs. One of the first groundbreaking efforts to develop CHW competencies came in 1998 with the report from the National Community Health Advisor Study (23). Another effort came in the form of a strategic initiative of the California Endowment's Building Healthy Communities partnering with the organization *Vis*í*on y Compromiso*, which resulted in a framing paper highlighting roles and 10 defining characteristics and values that make a successful *promotora de salud* (24). This effort emphasized the importance of CHW qualities of having similar life experiences as the community being served, being trusted members of the community, and communicating community needs to organizations and compassion-based service, describing it as “*servicio de corazón*” (service from the heart) (24). A more recent research initiative attempted to establish a validated, standardized set of 27 core CHW competencies and a linked workforce framework, delineating three categories of CHWs based upon training, workplace, and scope of practice (25). However, this effort has been critiqued by the National Association of Community Health Workers (NACHW) because it “. relied on a small sample of primarily clinically based CHWs, (which) resulted in an overly medicalized model of core competencies that is inadequately aligned with CHW workforce history, current practice, and well-regarded research” (26). The most comprehensive and widely accepted set of CHW standards to date was proposed by The CHW Core Consensus Project (The C3 Project), a nationally-based collaborative effort between working CHWs, CHW curriculum developers and other allies (27). The C3 project proposed a recommended list of 10 roles and 11 skills and endorsed existing knowledge about CHW qualities that may be used as a reference for working CHWs or those working with CHWs. The concept of “qualities” allows for capturing what is already known about who makes a good CHW, as prominently defined in the *American Public Health Association* CHW Section's definition of a CHW. One of the fundamental qualities in this definition is “[being] a trusted member of and/or [having] an unusually close understanding of the community served” (28). Such a close connection to the community served can facilitate not only trust with patients, but also the CHWs' ability to better communicate with patients as well as serve as bridges of communications between clinical health care teams and such patients.

While CHW roles and competencies have been proposed, there are no formalized frameworks or standards-based curricula reported in the literature for training clinical CHWs in the United States. In our estimation, a remaining gap in the literature is a published account of a framework and description of applying proposed standards and competencies to develop a comprehensive training for CHWs in clinical settings. To the best of our knowledge, this is the first such effort to do so. While we are familiar with the existence of other well-regarded training programs for clinical CHWs such as Loma Linda University's San Manuel Gateway College Promotores Academy and University of Pennsylvania's Penn Center for Community Health Workers, we do not know of any publications on the development of such a training curriculum for clinically based CHWs. The Charles R. Drew University (CDU) CHW Academy is committed to addressing these gaps in the training and placement of CHWs from diverse backgrounds into clinical settings using an innovative approach to developing such curricula. In line with the C3 project, we see a distinction in how CHWs function when they are based in the community vs. in the clinic. Furthermore, they may have a different emphasis depending on their location and the primary context of their work. This can take several forms along a spectrum that includes either community-based or clinic-based CHWs who could be community-facing or clinic-facing. For our curriculum, we are focusing on CHWs who are clinic-based and community facing. But a strong element of our curriculum is that we have adhered to the C3 skills and competencies that are common to all CHWs. Below we discuss: (a) the conceptual approach to our curricular development and its innovative features; (b) the learning environment, objectives and format of our curriculum; (c) results to date, including our core curricular modules and evaluation plans; (d) practical implications and lessons learned; and (e) some historical, environmental, and material constraints that have shaped and limited our planned development.

## Conceptual Approach

**Figure 1:**
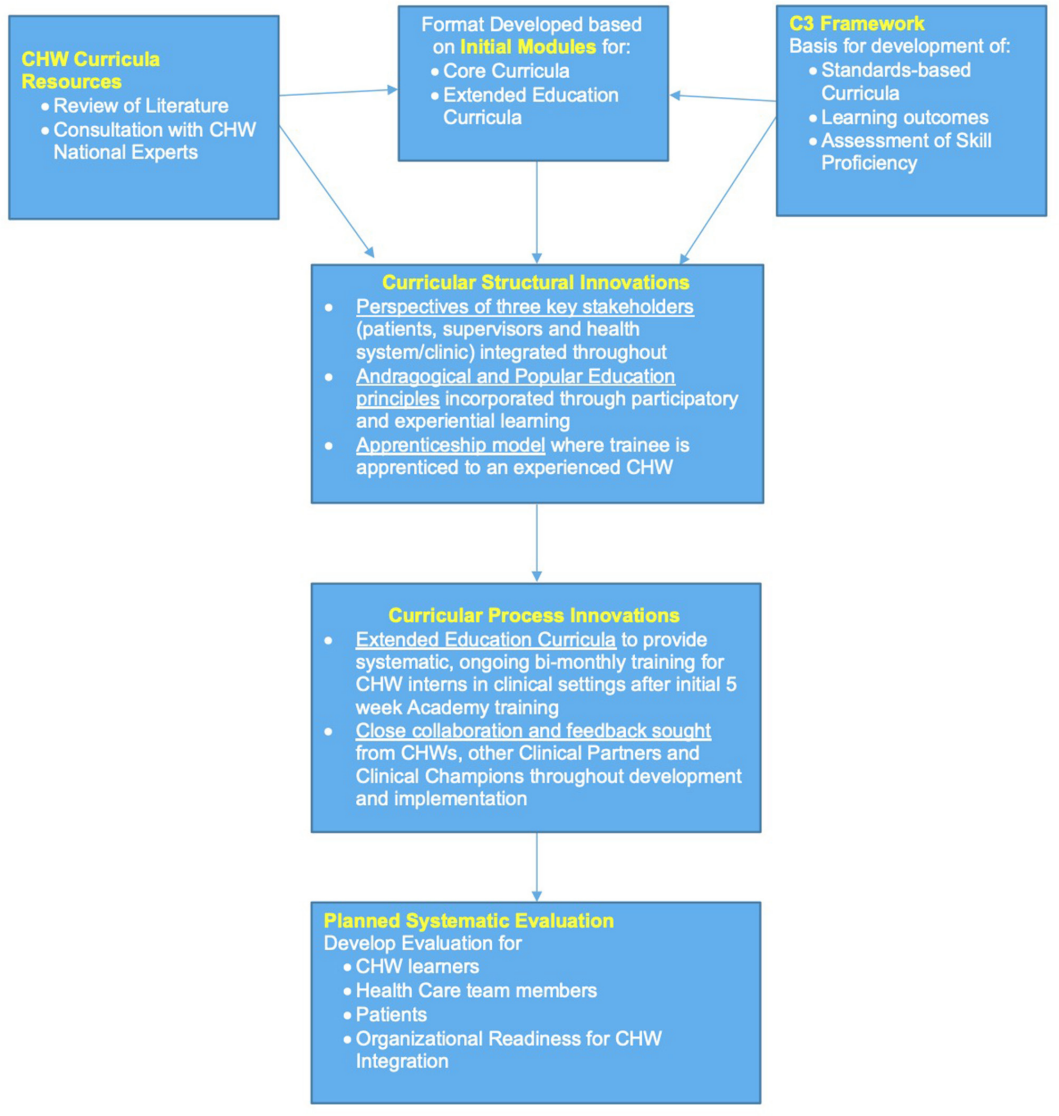
CDU CHW academy curriculum development framework.

The conceptual approach for our curricular development included several innovative features outlined in Figure 1. Below we identify six key features that grounded our curriculum development approach. *First*, we began this process with a literature review and a foundational consultation process with CHW national experts to inform our curricular development approach, process and content. This group included nationally recognized CHWs and CHW allies with a track record of expertise in research, policy, and advocacy on behalf of CHWs. Using reflections sent by our expert panel on a set of questions to guide our initial conceptualization of the curriculum, we held a 2 day in person retreat with the expert panel at CDU, where they advised us on our overall approach, the processes of development and implementation as well as insights on the planned content of the curriculum. *Second*, based on our review of the literature and input from the national experts, we used the CHW Consensus Project (C3 Project) to provide curricular standards and guide the identification of content areas and learning objectives for the 10 modules of our curriculum. The C3 project includes a list of 10 CHW roles/functions and 11 associated skills, with multiple skills and CHW qualities necessary to support each role (29). The 10 CHW roles proposed by the C3 project refer to key CHW functions related to (1) cultural mediation, (2) culturally-appropriate health education, (3) care coordination and navigation, (4) coaching/social support, (5) advocacy, (6) capacity building, (7) direct services (8) individual/community assessment, (9) outreach, (10) evaluation and research (29). The grassroots history of CHWs, their ability to catalyze community growth, and the necessity and dynamics of their communication skills are reflected in the list of the ten roles and eleven skills proposed by the C3 project (9). *Third*, we identified three key stakeholders—patients, health care teams, and health care systems—who would be affected by CHWs in the clinical setting. Thus, with each module, we integrated and incorporated the perspectives of each of these stakeholders throughout the curriculum. *Fourth*, our approach utilized popular education principles, which aim to foster a collaborative learning process. For example, we used participatory approaches that focus on problem solving and role playing, including hands-on laboratory sessions to practice skills. *Fifth*, in place of a pedagogy, we have used an andragogical approach that recognizes that our learners come with their own lived experiences. We integrated adult learning principles throughout our curriculum (e.g., more problem centered than content oriented), in how we conduct our assessments of the learners and in a culminating apprenticeship model of learning. *Sixth*, we incorporated hands on collaboration with our clinical partners throughout the stages of the planning and development of our curriculum. Two practicing CHWs and co-authors (IR and ML) working with our clinical partners reviewed each module and other aspects of our curriculum as we developed them and these CHWs provided feedback and input through track changed comments and participating in weekly discussion meetings on the curriculum. Furthermore, regular meetings over the past 2 years (quarterly or more often depending on the need) with our health care organization partners, who are also co-authors of this paper (JT, JJ, JM, RS), helped assure clinical relevance of our curriculum.

## Learning Environment, Objectives, and Format

**Figure 2:**
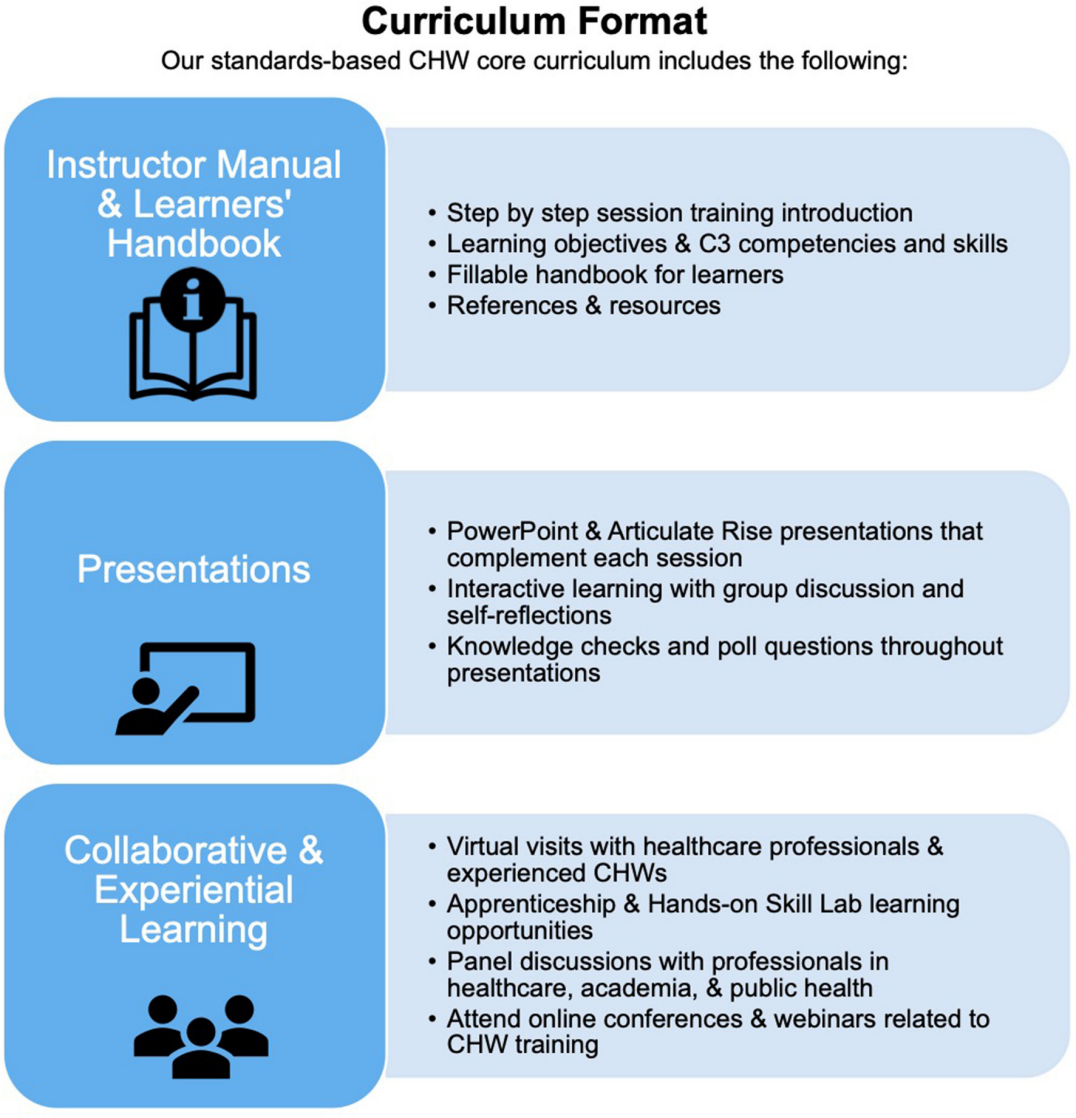
CDU CHW academy core curriculum format.

Our curriculum was planned in the context of a partnership between the *CDU CHW Academy* and a regional healthcare organization, *Providence*, with the intention of training CHWs to be placed in regional clinics and hospitals. We have recruited our cohorts of students from community settings surrounding the clinics across Los Angeles County where we intend to place the trained CHWs. Our CDU CHW Academy Instructors, coming from the same communities as our students, are also the curriculum developers under the direction of the two Academy directors. They are supported by five student interns at various stages of their health professional trainings. The learning objectives for each module was developed by our team based on guidance provided by C3 standards as well as use of the CHW Foundations textbook as a resource (30). Our pedagogical approach, or rather our andragogical approach, is outlined in Figure 2. The figure illustrates the multiple formats we use to present our curricular content for both in person and online implementation. First, learners are provided a student handbook with fillable exercise pages and course materials that match the instructor's more extensive textbook. Second, course materials are presented using both PowerPoint slides and Articulate 360, a web-based course authoring software that allows for dynamic and interactive presentation of materials and engaging assessment of learners' comprehension through knowledge check and poll questions. Finally, in keeping with the application of popular education and andragogical principles, our curriculum emphasizes several interactive methods to engage the learners. For example, most didactic sessions last about 4 h, including lecture presentation, individual reflections, group chat discussions, poll questions, and small group work. Fifty percent of each day is spent in a Hands-on Lab to practice the roles and skills discussed in the module of focus. Daily skill labs will also last 4 h with interactive activities including role play, reflective journaling, process simulations, individual and group assessments, and independent work. Interspersed into these sessions will be panel discussions with professionals in health care, academia, and public health. Students will be given the opportunity to attend online conferences and webinars related to CHW training. Finally, we plan to implement a modified apprenticeship program through virtual visits with healthcare professionals and experienced CHWs, given the limitations of the current pandemic.

## Results-Curricular Modules and Evaluation Plans

**Figure 3:**
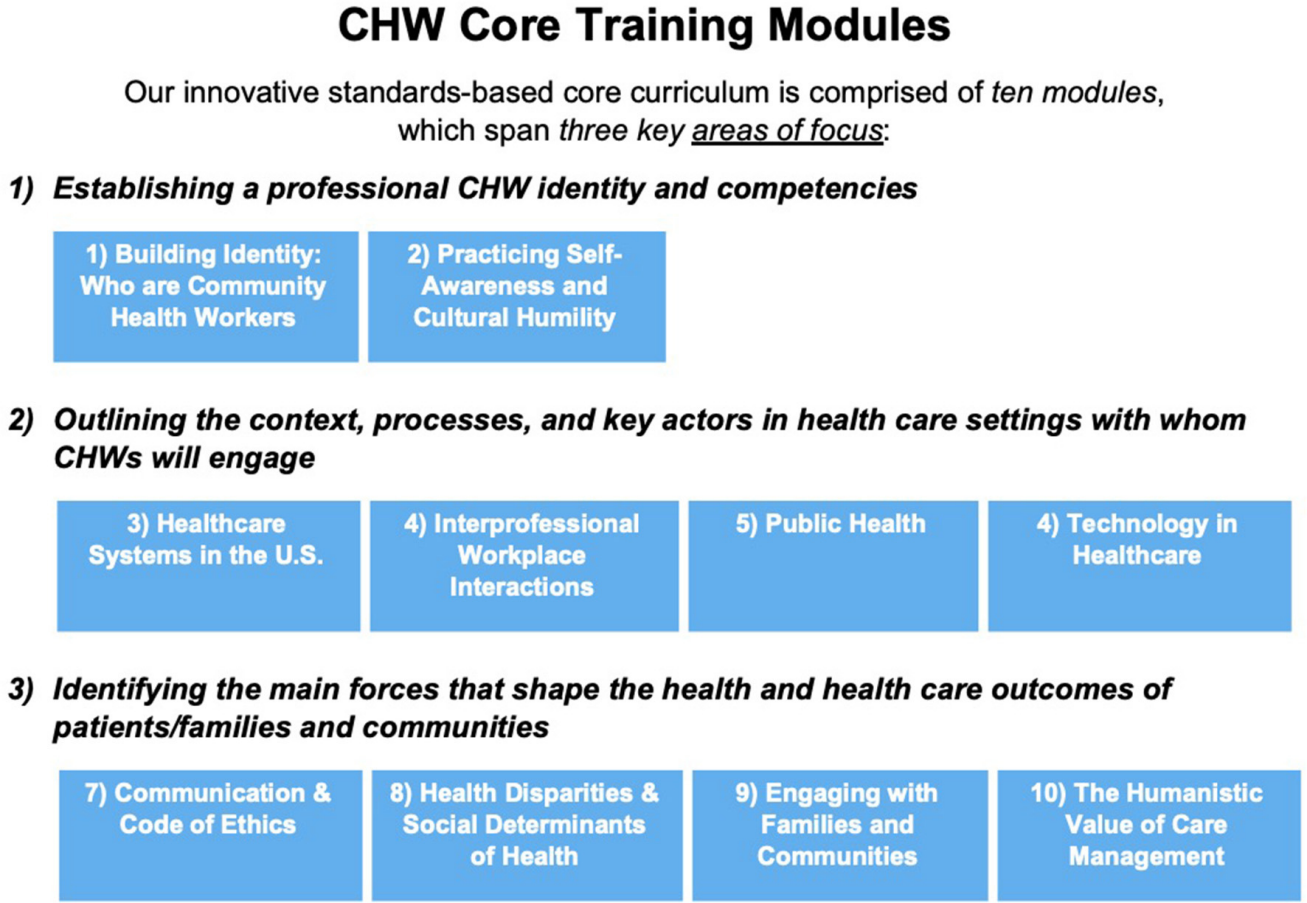
CDU CHW core training modules.

The resulting innovative standards-based curriculum is comprised of 10 modules, which span three key areas of focus as outlined in Figure 3. Each of the 10 modules include two didactic sessions and associated lab sessions that allow learners to engage and practice and further explore new competencies introduced in each module. Each session will include the following structure: (1) Introduction and review of learning objectives, (2) content presentation, (3) teach back and summary, (4) skills review and chapter check, and (5) closing and answering remaining student questions. Furthermore, in Supplementary Material 1, we list the specific C3 standards that have been addressed through the content and learning objectives for each of the 10 modules of our curriculum.

As outlined in Figure 3, the first area of focus in these modules is related to “*Establishing a professional CHW Identity”* which consists of modules one and two. We assume that most of the students will have no previous experience or knowledge about CHWs. Consequently, in the first module, we begin with an introduction to CHWs, their legacy and impact in the community and health care settings. We also introduce the learners to the C3 roles and competencies. Finally, we make them aware of the different CHW professional organizations and networks that exist to increase awareness and advocacy for the profession. In the second module, we focus on the importance of CHWs practicing self-awareness and cultural humility. For CHWs who are front line providers, it is especially important to be aware of signs of stress in themselves and develop an action plan to address such stress. We also introduce them to the concept of culture, the importance of cultural identities and culturally-based health beliefs, and the impact of cultural diversity among patients and co-workers on CHW work interactions and practices. As they reflect on their own cultural identities and associated health beliefs, we discuss the principles of cultural humility and how to apply these principles in their professional interactions.

The second area of focus is related to “*Outlining the context, processes and key actors in health care settings with whom CHWs will engage,”* which consists of modules three to seven. In the third module, we introduce the students to the U.S. health care systems in a comparative global context as well as in the context of the triple aims of improving the experience of care, improving the health of populations, and reducing per capita costs of health care. We do this by identifying four key players (patients, providers, payors, and policymakers), the differences between private and public insurance programs and different types of hospital systems (private, public, and non-profit). We also discuss the navigation of the health care system from the perspective of patients receiving different types of patient care (primary care, specialty care, and emergency care), the typical barriers patients may encounter when accessing health care services and the many ways that CHWs can facilitate such access and support navigation for patients. In the fourth module, we focus on interprofessional workplace interactions. We begin this module with a description of the health care team approach, the various types and roles of health care professionals on such health care teams, and the traits of successful multiprofessional health care teams. We focus on the role of CHWs on such teams as navigators, advocates, care coordinators, health promoters, etc. We also identify common barriers of interprofessional teamwork and discuss how they impact CHWs and how they may be addressed through conflict resolution principles to help CHWs be more effective members of health care teams. In the fifth module, we introduce learners to the field of public health, its emphasis on population, prevention, and social justice and its three main functions of assessment, policy development, and assurance. We describe how the three levels of public health departments at the federal, state, and local levels can work in tandem with the field of medicine, and the roles that CHWs can play in prevention initiatives, using the COVID 19 pandemic and contact tracing as examples. We also discuss how public health is a multidisciplinary field, rooted in epidemiology and community health, using the ecological model to illustrate a public health approach to addressing population health. In the sixth module, we address the existing, new, and expanding uses of technology in health care. We first introduce students to the types of technology used in health care and how they are used by providers and patients. We describe some of the benefits and challenges of telehealth-based health care provision and discuss ways to protect privacy and confidentiality when using online applications. We consider how CHWs may build trust with patients in virtual settings and illustrate this through examples of how to build trusting relations with patients, families, and community members. In the seventh module, we focus on the vital importance of communication skills as well as ethical and legal regulations for CHW professional interactions. First, we Identify methods to communicate with patients and health care teams effectively, using verbal, non-verbal, and written communication skills. We analyze the value of and strategies for both providing and receiving constructive feedback in employment settings. We define code switching and potential challenges as well as discuss strategies for how and when to switch codes while retaining one's personal identity. We end this module by introducing legal regulations that operate in clinical settings, such as The Health Insurance Portability and Accountability Act (HIPPA) and the need for informed consent vs. ethics as guiding principles for CHWs and how they are different from laws. We discuss the Framework for Ethical Decision Making and key articles from the CHW Code of Ethics. While we provide examples of applying such codes as ethical guidelines relating to informed consent and confidentiality, we also highlight limits on confidentiality, given the CHW role as a mandated reporter.

The third area of focus is related to “*Identifying the main forces that shape the health and health care outcomes of patients/families and communities,”* consisting of modules eight to ten. In the eighth module, we introduce students to health disparities and social determinants of health. We specifically address health disparities related to race/ethnicity, gender/sex, and socioeconomic status, resulting in inequity in access to care as well as, affordability and quality of care. We list and discuss the nine social determinants of health described by the World Health Organization, their influence on health outcomes and measurement tools commonly used by CHWs to assess social determinants of health. Finally, we outline the difference between health equality and health equity and discuss the goals of federal health policy agenda *Healthy People 2020* to promote health equity as well as how CHWs can play a role in helping address health disparities and the social determinants of health. In the ninth module, we examine social support resources to engage with families and communities. We begin with a discussion of the diverse range of families that CHWs are likely to encounter and ways in which CHWs may stay actively engaged with the patient's family members, while being mindful of the patient's particular culture (e.g., *familismo*) and family structure (e.g., extended families living together), using health promotion and treatment frameworks that incorporate the family. We focus on the “home visit,” a key tool for CHWs in engaging patients and provide examples and case studies of why and how they are conducted. Extending from the family to community, we discuss community engagement, organizing and advocacy and the CHW's potential role in the community capacity building. We introduce students to various models of community engagement such as Community Based Participatory Research (CBPR) and Community Action Model (CAM). In addition, we introduce them to key social services and how they might support patients in accessing and enrolling in such services. Finally in the 10 module, we conclude with a reflection on how all the CHW's roles and competencies culminate in the higher goal of ensuring humanistic values in care management. We begin by defining and illustrating humanistic values in health care through examples in health care settings. Reflecting on the potential challenges of a patient, who may have low health literacy and possible history of mistrust, and challenges navigating the health care system, we consider how a CHW might bring humanistic values to such a patient's experience of health care. We also focus on the CHW's role of care coordination, first considering varying definitions of care coordination and providing illustrations through examples of how CHWs engage with patients and collaborate with health care teams to coordinate care. We explore three key tools in the CHW care coordination toolkit of (1) identifying the social determinants of health at play in the patient's health situation, (2) using empowerment approaches to manage care, and (3) applying the various functions of the CHW role to manage the patient's care.

We have also planned a systematic evaluation of the curriculum implementation process. We will assess the learning outcomes of CHW students with pre- and post-written and oral rubric-based assessments that match the learning outcomes for each module. Additionally, we will employ both individual and group interactive self and peer assessments throughout the 5 weeks of curriculum implementation, keeping in line with popular education and andragogical principles. We hope to eventually expand our evaluation to the health care team members and health systems where our CHWs are placed as well as patients in the health system who receive care from CHWs. Our goal is to use this information to develop an organizational readiness program to better prepare our clinical internship host partners to best support the integration of CHWs into their health care teams and health systems.

## Discussion On the Practical Implications and Lessons Learned

We highlight four lessons from our curriculum development process that may help other such efforts. *First*, we have learned that it is important to review and build on (a) existing knowledge in the literature, (b) previous efforts in the development of CHW training curricula, and (c) the values of the community who are going to be the end users and beneficiaries of such an effort. We began with an extensive review of the literature which helped us frame our preliminary framework and questions. We sought the input of trusted nationally recognized experts in the field with many decades of work with CHWs, including some CHWs and CHW allies, clinical, and academic partners whose wisdom was invaluable to our process. With their guidance, we did not have to “reinvent the wheel,” but were directed to existing resources, some of which we used and adapted to develop our curriculum. Thus, we were able to sift through available materials to identify the C3 CHW standards as the basis on which to establish our curricular content. We also found the exemplary CHW Foundations textbook (30) to be extremely helpful and we have selected some of their materials, and adapted others to fit the clinical setting. All through this process, we remained aware of the community-focused principles of this field that were developed over many decades to inform our curricular content and learning outcomes.

**Figure 4:**
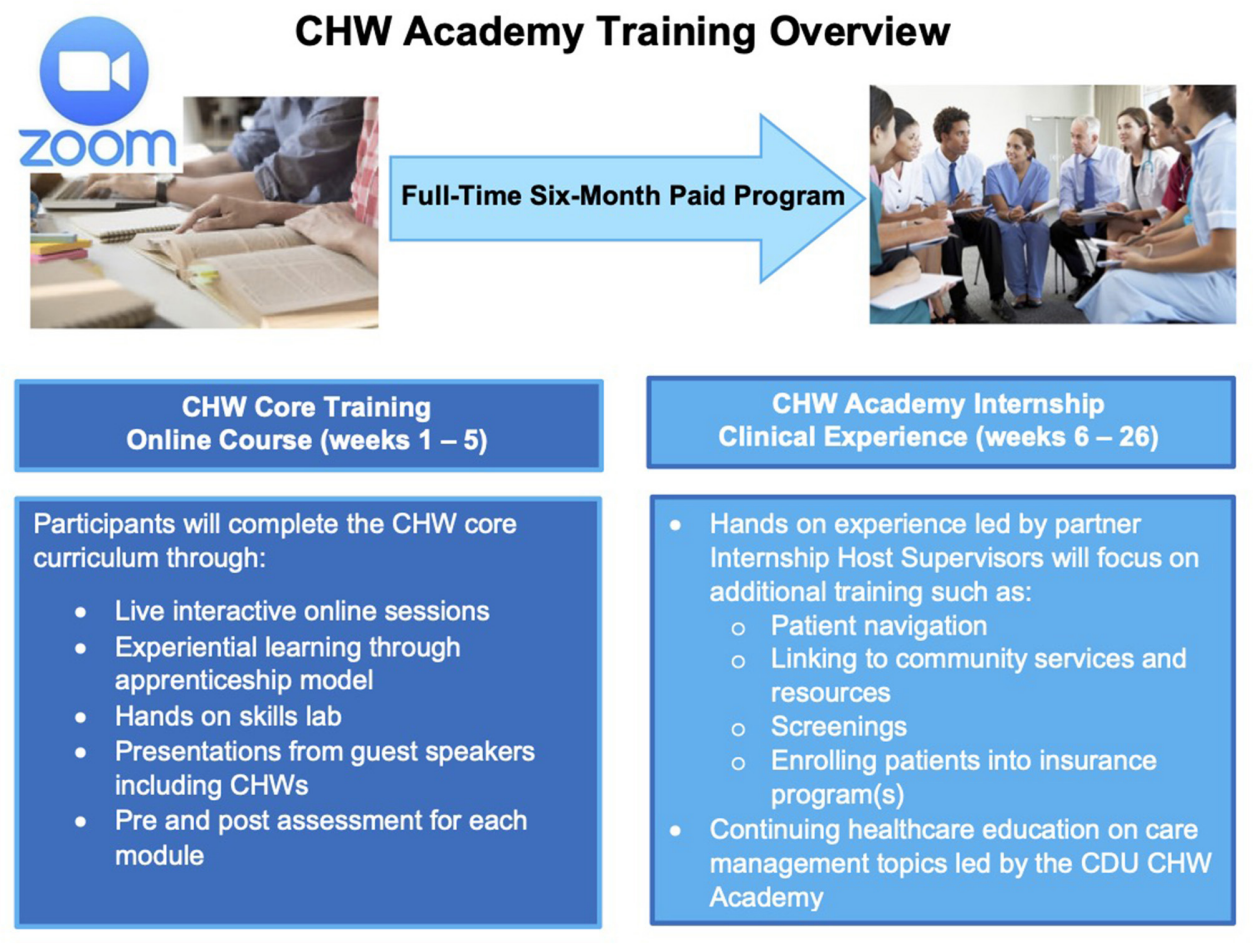
CDU CHW academy training overview.

*Second*, we have learned that curricula should support training delivery using experience-based, participatory approaches, consistent with adult education and popular education principles. Given our own previous experiences in curricular development and education as well as our work with CHWs, we understand the importance of meeting students where they are, especially adult learners with multiple competing demands on their attention. Furthermore, many of these adult learners come with rich lived experiences that can be leveraged to engage them and make the educational process more relevant for them. Finally, some of these students may come with lower literacy levels, learning disabilities and other challenges where interactive, participatory approaches are important in both the provision and assessment of curricular materials. In the spirit of experiential learning, our approach includes an internship opportunity for CHW students, as outlined in Figure 4. Thus, our students will not only have the 5 weeks of core training with us but will also participate in a 21 week fully paid internship in clinical settings where they will receive oversight and additional training as part of a modified apprenticeship program.

*Third*, we have learned that CHW training development for clinical settings should be built on a strong academic-clinical partnership, especially drawing on clinical CHWs and other clinical partner experiences. We were able to get input from our clinical partner administrators, and CHWs working in our partner health system all along the way in the development of our curricular plan and on each of the modules of our curriculum. In return, we have provided feedback on the internship plan and recruitment process of the student cohorts that is being managed by our clinical partners. Such a strong and reciprocal engagement and feedback from both academic and clinical partners, particularly CHWs, was invaluable to developing CHW training materials that are relevant to the clinical setting.

*Fourth*, we have learned that the curricular approach should ideally support training for key stakeholders and champions in clinical organizations to improve organizational readiness for integrating CHWs into healthcare teams and health systems. We have learned the importance of organizational readiness from the work done by our clinical partners in readying other care organizations in taking on our CHW students as interns in their settings. One of our clinical partner team members dedicated a considerable portion of her effort in cultivating the idea of CHWs as members of health care teams with our internship host sites. She identified clinical providers who could be champions of CHWs in these settings and is engaging them in regular meetings to support the integration and supervision of CHWs in health care teams. We also planned a series of meetings, including a “kickoff” meeting with the health care organizations' leadership, to introduce them to the development and implementation of this curriculum and the internship process.

## Conclusion

We hope that our results contribute to the growing research on effective CHW training methods and provide guidance to CHW training development for clinical settings. This was an especially difficult year in which to develop and plan for implementation of this curriculum, given the global COVID 19 pandemic and given that we were developing training for frontline healthcare workers. Thus, there are some COVID related limitations to our planned process. For example, while we hoped to implement a full-fledged apprenticeship model partnering CHW learners with experienced clinical CHWs, we had to modify the apprenticeship to limited engagement with clinical CHWs, given the unprecedented challenges faced by all clinical providers. Similarly, we had to adapt the curriculum content to be delivered exclusively online as a necessary adjustment to a pandemic environment. As we move past the pandemic, we hope that we will be able to implement a more comprehensive apprenticeship model and continuing education as a standardized part of our training approach and be able to provide an in person or hybrid approach to curriculum delivery. In order to address concerns raised by how standardizing CHW training can affect the emphasis on maintaining CHW community identity so crucial to their practice, in our training, we have focused on the related concepts of an ecological approach, the social determinants of health, cultural identity and health beliefs, and community engagement. All throughout our curriculum, we have emphasized and illustrated through examples how paying attention to the patients' broader socioeconomic and cultural context at the community level is vitally important not only to patient health outcomes, but also to bringing the full impact of the CHW role into clinical settings. We also recognize that the model we use of first recruiting and then training CHWs is not the traditional model of CHW workforce recruitment. Rather than engaging organically existing community health workers from the community, we are identifying individuals who may not have had previous experience in bridging the gap between clinical settings and the communities they live in and training them to do this work. It may be important to evaluate this non-traditional aspect of our approach in the future. We have yet to fully implement and evaluate our curriculum. We hope that our implementation and evaluation experiences will feed into the development of a formal organizational readiness component. Such an additional component, we hope, can be used to better prepare our clinical partners to engage and integrate CHWs fully into their healthcare teams and settings and to better fulfill the CHWs' potential to be bridges between patients, communities, healthcare providers, healthcare teams, and health systems.

## Data Availability Statement

The original contributions presented in the study are included in the article/Supplementary Material, further inquiries can be directed to the corresponding author/s.

## Author Contributions

SG and HB devised the main conceptual ideas of the project with substantial feedback from our clinical partners, JT, JJ, and JM. LS developed and worked out almost all of the technical details related to the curriculum with the support of RS and feedback from ML and IR. TV, and SN supported the development of background and literature review for the paper. SG drafted the initial manuscript. All authors reviewed and approved the final manuscript.

## Conflict of Interest

The authors declare that the research was conducted in the absence of any commercial or financial relationships that could be construed as a potential conflict of interest.
