# Supplementary material for: The Development of a Novel, Standards-Based Core Curriculum for Community Facing, Clinic-Based Community Health Workers
Source: Front Public Health. 2021 Jun 7;9:663492. doi: 10.3389/fpubh.2021.663492 (PMC8215713; doi:10.3389/fpubh.2021.663492)
Supplement: Supplementary file 1 [file Data_Sheet_1.PDF]

Figure 4 - Integration of C3 standards into Learning Outcomes for each module

Modules & Sessions	Learning Outcomes	C3 Competencies	C3 Role(s)
Module #1: **Building Identity of Community Health Workers (CHWs)**<br><br>Session 1:<br><br>**Introducing Community Health Workers**	• Describe Community Health Workers (CHWs) and what they do<br>• Understand the legacy of CHWs and their impact around the world<br>• Describe the CHW workforce and networks of associations<br>• Define health and the seven dimensions of health<br>• Explain the role of patients, health care teams, health care systems<br>• Identify the personal qualities, roles, and competencies	• Use language confidently (Skill #1a)<br>• Work as a team member (Skill #2d)<br>• Facilitate group discussions and decision-making (Skill #6c)<br>• Seek out appropriate information and respond to questions about pertinent topics (Skill #6e)<br>• Find and share requested information (Skill #6f)<br>• Apply critical thinking techniques and problem solving (Skill #9c)<br>• Use pertinent technology (Skill #9d)<br>• Pursue continuing education and life-long learning opportunities (Skill #9e)<br>• Participate in professional development of peer CHWs and in networking among CHW groups (Skill #9i)<br>• Knowledge about health behavior theories (Skill #11d)<br>• Knowledge about mental/behavioral health issues and their connection to physical health (Skill #11e)<br>• Understand the communities CHWs serve (Skill #11g)	• Building Individual and Community Capacity: Building individual capacity (Role #6a) Training and building individual capacity with peers and among CHW groups (Role #6c)
Module #1: **Building Identity of Community Health Workers (CHWs)**<br><br>Session 2:<br><br>**The Roles and Competencies of CHWs**	• Describe qualities needed to be a successful CHW<br>• Discuss and identify the CHW Core Consensus Project (C3) roles & competencies<br>• List and discuss the C3 skills required for CHW roles of outreach provider, care manager, health educator, liaison, and data manager<br>• Identify personal strengths and areas improvement for CHW skills	• Use language confidently (Skill #1a)<br>• Ability to provide coaching and social support (Skill #2a)<br>• Work as a team member (Skill #2d)<br>• Use empowering and learner-centered teaching strategies (Skill #6a)<br>• Use a range of appropriate and effective educational techniques (Skill #6b)	• Providing Culturally Appropriate Health Education and Information: Educating individuals & communities about how to use health and social service systems (including understanding health and social services operate) (Role #1a)<br>• Health Education: Conducting health promotion and disease prevention education in a manner that matches linguistic

	of outreach provider, care manager, health educator, liaison, and data manager	• Facilitate group discussions and decision-making (Skill #6c)<br>• Seek out appropriate information and respond to questions about pertinent topics (Skill #6e)<br>• Find and share requested information (Skill #6f)<br>• Apply critical thinking techniques and problem solving (Skill #9c)<br>• Use pertinent technology (Skill #9d)<br>• Pursue continuing education and life-long learning opportunities (Skill #9e)<br>• Participate in professional development of peer CHWs and in networking among CHW groups (Skill #9i)	and cultural needs of participants or community (Role #2a) Providing necessary information to understand and prevent diseases and to help people manage health conditions (including chronic diseases) (Role #2b)<br>• Care Management & Navigation: Participating in care coordination and/or case management (Role #3a) Making referrals & providing follow-up (Role #3b) Facilitating transportation to services & helping address barriers to services (Role #3c)<br>• Building Individual and Community Capacity: Training and building individual capacity with peers and among CHW groups (Role #6c)<br>• Outreach: Case finding/ recruitment of individuals, families, and community groups to services and systems (Role #9a)<br>Evaluation: Engaging in evaluating CHW services and programs (Role #10a)
Module #2: **Self-Awareness and Cultural Humility**<br>Session 1:<br>**Developing Self-Awareness and Self-Care**	• Understand the importance of self-awareness among CHWs<br>• Describe ways to increase self-awareness<br>• Identify personal values and beliefs that may influence the ability to provide patient care<br>• Describe and assess personal signs of stress<br>• Develop an action plan for self-care	• Use language confidently (Skill #1a)<br>• Use language in ways that engage and motivate (Skill #1b)<br>• Work as a team member (Skill #2d)<br>• Facilitate group discussions and decision-making (Skill #6c)<br>• Seek out appropriate information and respond to questions about pertinent topics (Skill #6e)<br>• Find and share requested information (Skill #6f)<br>• Set goals and to develop and follow a work plan (Skill #9a)<br>• Apply critical thinking techniques and problem solving (Skill #9c)	• Coaching and Social Support: Provide individual support and coaching (Role #4a)<br>• Building Individual and Community Capacity: Train and build individual capacity with peers and among CHW groups (Role #6c)

		• Use pertinent technology (Skill #9d)<br><br>• Pursue continuing education and life-long learning opportunities (Skill #9e)<br><br>• Set boundaries and practice self-care (Skill #9j)<br><br>• Participate in professional development of peer CHWs and in networking among CHW groups (Skill #9i)<br><br>• Understand and identify healthy lifestyles and self-care (Skill #11c)<br><br>• Knowledge about mental/behavioral health issues and their connection to physical health (Skill #11e)	
Module #2: **Self-Awareness and Cultural Humility**<br><br><u>Session 2:</u><br><br>**The Importance of Culture and Cultural Humility**	• Discuss concepts of culture<br><br>• Reflect upon their own cultural identities<br><br>• Describe traditional health beliefs and their influence on client services<br><br>• Discuss the principles of cultural humility<br><br>• Identify and apply the LEARN model for practicing cultural humility<br><br>• Understand the value of being lifelong learners and practitioners of cultural humility	• Use language confidently (Skill #1a)<br><br>• Communicate with the communities they serve (may not be fluent in the language of all communities) (Skill #1h)<br><br>• Work as a team member (Skill #2d)<br><br>• Practice cultural humility (Skill #2f)<br><br>• Work in ways that increase individual and community empowerment (Skill #4b)<br><br>• Use empowering and learner-centered teaching strategies (Skill #6a)<br><br>• Use a range of appropriate and effective educational techniques (Skill #6b)<br><br>• Facilitate group discussions and decision-making (Skill #6c)<br><br>• Seek out appropriate information and respond to questions about pertinent topics (Skill #6e)<br><br>• Find and share requested information (Skill #6f)<br><br>• Apply critical thinking techniques and problem solving (Skill #9c)<br><br>• Use pertinent technology (Skill #9d)	• <u>Providing Culturally Appropriate Health Education and Information:</u> Educating systems about community perspectives and cultural norms (including supporting implementing of Culturally and Linguistically Appropriate Services [CLAS] standards) (Role #1b)<br><br>• <u>Building Individual and Community Capacity</u> Building individual capacity (Role #6a) Training and building individual capacity with peers and among CHW groups (Role #6c)

		• Pursue continuing education and life-long learning opportunities (Skill #9e)<br>• Participate in the professional development of peer CHWs and in networking among CHW groups (Skill #9i)<br>• Knowledge about health behaviors theories (Skill #11e)	
Module #3: **Health care Systems in the U.S.**<br>Session 1:<br>**The U.S. Health Care System**	• Discuss how patients, providers, payors, and policymakers are the four key parts of the U.S. health care system<br>• Describe who provides health care services in the U.S.<br>• Describe the differences between private, public, and non-profit hospital systems<br>• Identify types of private insurance plans and public insurance programs<br>• Describe the health status in the U.S. compared to other nations	• Use language confidently (Skill #1a)<br>• Work as a team member (Skill #2d)<br>• Coordinate CHW activities with clinical and other community services (Skill #3d)<br>• Follow-up and track care and referral outcomes (Skill #3e)<br>• Facilitate group discussions and decision-making (Skill #6c)<br>• Seek out appropriate information and respond to questions about pertinent topics (Skill #6e)<br>• Find and share requested information (Skill #6f)<br>• Apply critical thinking techniques and problem solving (Skill #9c)<br>• Use pertinent technology (Skill #9d)<br>• Pursue continuing education and life-long learning opportunities (Skill #9e)<br>• Participate in professional development of peer CHWs and in networking among CHW groups (Skill #9i)<br>• Knowledge about United States health and social service systems (Skill #11h)	• Cultural Mediation Among Individuals, Communities, and Health and Social Service Systems: Educating individuals and communities about how to use health and social service systems (including understanding how systems operate) (Role #1a)<br>• Building Individual and Community Capacity: Train and build individual capacity with peers and among CHW groups (Role #6c)<br>• Advocating for Individual and Communities: Connecting to resources and advocating for basic needs (e.g. food and housing) (Role #5b)<br>• Providing Direct Service: Meeting basic needs (e.g. direct provisions of food and other resources) (Role #7c)
Module #3: **Health care Systems in the U.S.**<br>Session 2:<br>**Navigating the Health Care System**	• Define types of patient care including primary care, specialty care and emergency care<br>• Describe barriers patients may encounter when accessing health care services	• Use language confidently (Skill #1a)<br>• Provide coaching and social support (Skill #2a)<br>• Conduct self-management coaching (Skill #2b)<br>• Work as a team member (Skill #2d)	• Cultural Mediation Among Individuals, Communities, and Health and Social Service Systems Educating systems about community perspectives and cultural norms (including supporting implementation of Culturally and Linguistically Appropriate Services [CLAS] standards) (Role #1b) Building

	• Identify how CHWs can assist patients in overcoming barriers to health care<br>• Discuss health care access and navigation<br>• Describe how CHWs support the triple aim framework within the health care system	• Coordinate care (including identifying and accessing resources and overcoming barriers) (Skill #3a)<br>• Coordinate CHW activities with clinical and other community services (Skill #3d)<br>• Follow-up and track care and referral outcomes (Skill #3e)<br>• Use empowering and learner-centered teaching strategies (Skill #6a)<br>• Use a range of appropriate and effective educational techniques (Skill #6b)<br>• Facilitate group discussions and decision-making (Skill #6c)<br>• Seek out appropriate information and respond to questions about pertinent topics (Skill #6e)<br>• Find and share requested information (Skill #6f)<br>• Build and maintain a current resource inventory (Skill #8c)<br>• Apply critical thinking techniques and problem solving (Skill #9c)<br>• Use pertinent technology (Skill #9d)<br>• Pursue continuing education and life-long learning opportunities (Skill #9e)<br>• Participate in professional development of peer CHWs and in networking among CHW groups (Skill #9i)<br>• Knowledge about United States health and social service systems (Skill #11h)	health literacy and cross-cultural communication (Role #1c)<br>• Advocating for Individual and Communities: Connecting to resources and advocating for basic needs (e.g. food and housing) (Role #5b)<br>• Providing Direct Service: Meeting basic needs (e.g. direct provisions of food and other resources) (Role #7c)<br>• Building Individual and Community Capacity: Train and build individual capacity with peers and among CHW groups (Role #6c)
Module #4: **Interprofessional Workplace Interactions**<br>Session 1:<br>**Interprofessional Teamwork**	• Describe the health care team approach<br>• Describe the various types and roles of health care professionals<br>• Identify traits of successful health care teams	• Use language confidently (Skill #1a)<br>• Work as a team member (Skill #2d)<br>• Facilitate group discussions and decision-making (Skill #6c)<br>• Seek out appropriate information and respond to questions about pertinent topics (Skill #6e)	• Care Coordination, Case Management, and System Navigation: Inform people and systems about community assets and challenges (Role #3e)<br>• Building Individual and Community Capacity: Train and build individual capacity

	• Define and explain the terms interprofessional, collaboration, teams<br><br>• Describe the role of CHWs on such teams as the navigator, advocator, connector, and investigator	• Find and share requested information (Skill #6f)<br><br>• Apply critical thinking techniques and problem solving (Skill #9c)<br><br>• Use pertinent technology (Skill #9d)<br><br>• Pursue continuing education and life-long learning opportunities (Skill #9e)<br><br>• Participate in professional development of peer CHWs and in networking among CHW groups (Skill #9i)<br><br>• Knowledge about the United States health and social service system (Skill #11h)	with peers and among CHW groups (Role #6c)<br><br>• Evaluation and Research Skills: Participating in evaluation and research: Engaging with stakeholder to take action on findings (Role #10c)
Module #4: **Interprofessional Workplace Interactions**<br><br>Session 2:<br><br>**Barriers and Facilitators to Interprofessional Collaborations**	• Identify common barriers of interprofessional teamwork<br><br>• Discuss how some of these barriers impact CHWs<br><br>• Discuss key concepts related to conflict resolution<br><br>• Explain the key elements of effective health care teams<br><br>• Discuss strategies for successfully being part of health care teams	• Use language confidently (Skill #1a)<br><br>• Work as a team member (Skill #2d)<br><br>• Manage conflict (Skill #2e)<br><br>• Use empowering and learner-centered teaching strategies (Skill #6a)<br><br>• Use a range of appropriate and effective educational techniques (Skill #6b)<br><br>• Facilitate group discussions and decision-making (Skill #6c)<br><br>• Seek out appropriate information and respond to questions about pertinent topics (Skill #6e)<br><br>• Find and share requested information (Skill #6f)<br><br>• Collaborate with other educators (Skill #6g)<br><br>• Apply critical thinking techniques and problem solving (Skill #9c)<br><br>• Use pertinent technology (Skill #9d)<br><br>• Pursue continuing education and life-long learning opportunities (Skill #9e)<br><br>• Participate in professional development of peer CHWs and in	• Care Coordination, Case Management, and System Navigation: Inform people and systems about community assets and challenges. (Role #3e)<br><br>• Building Individual and Community Capacity: Train and build individual capacity with peers and among CHW groups (Role #6c)<br><br>• Evaluation and Research Skills: Participating in evaluation and research: Engaging with stakeholder to take action on findings (Role #10c)

		networking among CHW groups (Skill #9i)	
Module #5: **Public Health**<br><br>Session 1:<br><br>**Understanding the Field of Public Health**	• Define public health and how CHWs work within public health<br>• Discuss public health's emphasis on population, prevention, and social justice<br>• Describe the ecological model of public health and apply it to specific public health issues<br>• Identify organizations and people promoting public health<br>• Explain how the field of public health is different from the field of medicine<br>• Describe how public health is rooted in scientific methods like epidemiology	• Use language confidently (Skill #1a)<br>• Work as a team member (Skill #2d)<br>• Coordinate CHW activities with clinical and other community services (Skill #3d)<br>• Work in ways that increase individual and community empowerment (Skill #4b)<br>• Speak up for individuals and communities (Skill #5c)<br>• Facilitate group discussions and decision-making (Skill #6c)<br>• Seek out appropriate information and respond to questions about pertinent topics (Skill #6e)<br>• Find and share requested information (Skill #6f)<br>• Apply critical thinking techniques and problem solving (Skill #9c)<br>• Use pertinent technology (Skill #9d)<br>• Pursue continuing education and life-long learning opportunities (Skill #9e)<br>• Participate in professional development of peer CHWs and in networking among CHW groups (Skill #9i)<br>• Knowledge about mental/behavioral health issues and their connection to physical health (Skill #11e)<br>• Understand basic public health principles (Skill #11f)<br>• Understand the communities served by CHWs (Skill #11g)	• Cultural Mediation Among Individuals, Communities, and Health and Social Service Systems Building health literacy and cross-cultural communication (Role #1c)<br>• Building Individual and Community Capacity: Building individual capacity (Role #6a) Training and building individual capacity with peers and among CHW groups (Role #6c)
Module #5: **Public Health**<br><br>Session 2:<br><br>**The Practice of Public Health**	• Describe the three main functions of public health department which include assessment, policy development, and assurance<br>• Identify and describe the three levels of public health department at federal, state, local levels	• Use language confidently (Skill #1a)<br>• Work as a team member (Skill #2d)<br>• Work in ways that increase individual and community empowerment (Skill #4b)	• Advocating for Individuals and Communities: Advocating for the needs and perspectives of communities (Role #5a) Connecting to resources and advocating for basic needs (Role #5b) Conducting policy advocacy (Role #5c)

	• Discuss how CHWs fit into the model of public health and prevention initiatives<br>• Describe the basic steps of contact tracing<br>• Identify the people involved in the contact tracing process<br>• Understand how contact tracing helps individuals, communities, and public health efforts	• Network, build community connections, and build coalitions (Skill #4c)<br>• Teach self-advocacy skills (Skill #4d)<br>• Contribute to policy development (Skill #5a)<br>• Advocate for policy change (Skill #5b)<br>• Speak up for individuals and communities (Skill #5c)<br>• Use empowering and learner-centered teaching strategies (Skill #6a)<br>• Use a range of appropriate and effective educational techniques (Skill #6b)<br>• Facilitate group discussions and decision-making (Skill #6c)<br>• Seek out appropriate information and respond to questions about pertinent topics (Skill #6e)<br>• Find and share requested information (Skill #6f)<br>• Apply critical thinking techniques and problem solving (Skill #9c)<br>• Use pertinent technology (Skill #9d)<br>• Pursue continuing education and life-long learning opportunities (Skill #9e)<br>• Participate in professional development of peer CHWs and in networking among CHW groups (Skill #9i)<br>• Understand basic public health principles (Skill #11f)<br>• Understand the communities served by CHWs (Skill #11g)	• Building Individual and Community Capacity: Train and build individual capacity with peers and among CHW groups (Role #6c)
Module # 6: **Technology in Health Care**<br>Session 1:<br>**Understanding the Use of**	• List the types of technology used in health care and describe how they are used by providers and patients<br>• Describe some of the benefits of telehealth	• Use language confidently (Skill #1a)<br>• Prepare written communication including electronic communication (e.g. email or telecommunication device for the deaf) (Skill #1f)	• Building Individual and Community Capacity: Building individual capacity (Role #6a) Building community capacity (Role #6b) Training and building individual capacity

**Technology in Health Care**	• Describe some of the challenges of telehealth<br>• List ways to protect privacy and confidentiality when using online applications	• Document work (Skill #1g)<br>• Work as a team member (Skill #2d)<br>• Facilitate group discussions and decision-making (Skill #6c)<br>• Seek out appropriate information and respond to questions about pertinent topics (Skill #6e)<br>• Find and share requested information (Skill #6f)<br>• Apply critical thinking techniques and problem solving (Skill #9c)<br>• Ability to use pertinent technology (Skill #9d)<br>• Ability to pursue continuing education and lifelong learning opportunities (Skill #9e)<br>• Participate in professional development of peer CHWs and in networking among CHW groups (Skill #9i)	with peers and among CHW groups (Role #6c)
Module # 6: **Technology in Health Care**<br>Session 2:<br>**Challenges and Opportunities of Technology in Health Care**	• Describe the challenges encountered in health information technology<br>• Explain what a CHW does to protect patient privacy<br>• Describe the Health Footprint of the Pandemic Framework to understand how COVID-19 has impacted patient care<br>• Discuss how CHWs may build trust with patient in a virtual setting<br>• Identify examples of how to build trusting relations with patients, families, and community members	• Use language confidently (Skill #1a)<br>• Document work (Skill #1g)<br>• Communicate with the community served (may not be fluent in language of all communities served) (Skill #1h)<br>• Work as a team member (Skill #2d)<br>• Use empowering and learner-centered teaching strategies (Skill #6a)<br>• Use a range of appropriate and effective educational techniques (Skill #6b)<br>• Facilitate group discussions and decision-making (Skill #6c)<br>• Seek out appropriate information and respond to questions about pertinent topics (Skill #6e)<br>• Find and share requested information (Skill #6f)<br>• Apply critical thinking techniques and problem solving (Skill #9c)<br>• Use pertinent technology (Skill #9d)	• Building Individual and Community Capacity: Building individual capacity (Role #6a) Building community capacity (Role #6b) Training and building individual capacity with peers and among CHW groups (Role #6c)

		• Pursue continuing education and life-long learning opportunities (Skill #9e)<br>• Observe ethical and legal standards Health Insurance Portability and Accountability Act, HIPAA (Skill #9g)<br>• Participate in professional development of peer CHWs and in networking among CHW groups (Skill #9i)	
Module # 7: **Communication Skills & Code of Ethics**<br><br><u>Session 1:</u><br><br>**Communication Skills for CHWs**	• Describe verbal, nonverbal, and written communication skills for CHWs<br>• Identify methods to communicate with patients and healthcare teams effectively<br>• Analyze the value of constructive feedback and the challenges to receiving feedback in a calm and respectful manner<br>• Identify strategies and skills for providing and receiving constructive feedback in employment settings<br>• Define code switching and potential challenges<br>• Discuss strategies for how and when to switch codes while retaining personal identities	• Use language confidently (Skill #1a)<br>• Use language in ways that engage and motivate (Skill #1b)<br>• Communicate using plain and clear language (Skill #1c)<br>• Communicate with empathy (Skill #1d)<br>• Listen actively (Skill #1e)<br>• Prepare written communication including electronic communication (e.g. email or telecommunication device for the deaf) (Skill#1f)<br>• Document work (Skill #1g)<br>• Communicate with the community served (may not be fluent in language of all community served) (Skill #1h)<br>• Use interviewing techniques (e.g. motivational interviewing) (Skill #1h)<br>• Work as a team member (Skill #2d)<br>• Facilitate group discussions and decision-making (Skill #6c)<br>• Seek out appropriate information and respond to questions about pertinent topics (Skill #6e)<br>• Find and share requested information (Skill #6f)<br>• Apply critical thinking techniques and problem solving (Skill #9c)<br>• Use pertinent technology (Skill #9d)	• <u>Cultural Mediation Among Individuals, Communities, and Health and Social Service Systems:</u> Building health literacy and cross-cultural communication (Role #1c)<br>• <u>Coaching and Social Support:</u> Motivating and encouraging people to obtain care and other services (Role #4b) Planning and/or leading support groups (Role #4d)<br>• <u>Building Individual and Community Capacity</u>: Training and building individual capacity with peers and among CHW groups (Role #6c)

		• Pursue continuing education and life-long learning opportunities (Skill #9e)<br>• Participate in professional development of peer CHWs and in networking among CHW groups (Skill #9i)	
Module # 7: **Communication Skills & Code of Ethics**<br><br><u>Session 2:</u><br><br>**Guiding Principles for CHWs**	• Describe HIPAA regulation and how CHWs may observe confidentiality and informed consent<br>• Explain the limits on confidentiality and role as a CHW mandated reporter<br>• Define ethics and explain how ethics are different from laws<br>• Discuss key articles from the CHW Code of Ethics<br>• Explain ethical guidelines relating to informed consent and confidentiality<br>• Apply the Framework for Ethical Decision Making to resolve ethical questions<br>• Define ethics and explain how ethics are different from laws<br>• Describe articles from the CHW Code of Ethics	• Use language confidently (Skill #1a)<br>• Work as a team member (Skill #2d)<br>• Use empowering and learner-centered teaching strategies (Skill #6a)<br>• Use a range of appropriate and effective educational techniques (Skill #6b)<br>• Facilitate group discussions and decision-making (Skill #6c)<br>• Seek out appropriate information and respond to questions about pertinent topics (Skill #6e)<br>• Find and share requested information (Skill #6f)<br>• Apply critical thinking techniques and problem solving (Skill #9c)<br>• Use pertinent technology (Skill #9d)<br>• Pursue continuing education and life-long learning opportunities (Skill #9e)<br>• Observe ethical and legal standards (e.g. CHW Code of Ethics, Americans with Disabilities Act [ADA], Health Insurance Portability, and Accountability Act [HIPAA]) (Skill #9g)<br>• Identify situations calling for mandatory reporting and carry out mandatory reporting requirements (Skill #9h)<br>• Participate in professional development of peer CHWs and in networking among CHW groups (Skill #9i)	• <u>Building Individual and Community Capacity</u>: Building individual capacity (Role #6a) Training and building individual capacity with peers and among CHW groups (Role #6c)
Module # 8: **Social Determinants of**	• Define health disparities<br>• Discuss evidence on health disparities	• Use language confidently (Skill #1a)<br>• Provide coaching and social support (Skill #2a)	• <u>Advocating for Individuals and Communities</u>: Advocate for the needs and perspectives of communities (Role #5a)

**Health and Health Equity**<br><br>Session 1:<br><br>**Health Disparities and Social Determinants of Health**	• Identify key factors that are associated with disparities in health such as race/ethnicity, gender/sex, and socioeconomic status<br><br>• Discuss disparities in health care by accessibility, affordability, and quality<br><br>• Analyze the impact of health disparities<br><br>• Describe social determinants of health and their influence on health outcomes<br><br>• Identify the nine social determinants of health described by the World Health Organization<br><br>• Identify assessment tools commonly used by CHWs to assess social determinants of health	• Work as a team member (Skill #2d)<br><br>• Work in ways that increase individual and community empowerment (Skill #4b)<br><br>• Facilitate group discussions and decision-making (Skill #6c)<br><br>• Seek out appropriate information and respond to questions about pertinent topics (Skill #6e)<br><br>• Find and share requested information (Skill #6f)<br><br>• Apply critical thinking techniques and problem solving (Skill #9c)<br><br>• Use pertinent technology (Skill #9d)<br><br>• Pursue continuing education and life-long learning opportunities (Skill #9e)<br><br>• Participate in professional development of peer CHWs and in networking among CHW groups (Skill #9i)<br><br>• Understand social determinants of health and related disparities (Skill #11a)<br><br>• Understand pertinent health issues (Skill #11b)<br><br>• Knowledge about mental/behavioral health issues and their connection to physical health (Skill #11e)<br><br>• Understand the communities CHWs serve (Skill #11g)<br><br>• Understand the United States health and social service systems (Skill #11h)	• Build Individual and Community Capacity: Build individual capacity (Role #6b) Build community capacity (Role #6a) Training and building individual capacity with peers and among CHW groups (Role #6c)
Module # 8:<br>**Social Determinants of Health and Health Equity**<br><br>Session 2:<br><br>**CHWs Promoting Health Equity**	• Define health equity<br><br>• Identify the difference between terms health equality and health equity<br><br>• Explain the role of Healthy People 2020 and their goals to promote health equity<br><br>• Describe how CHWs play a role in eliminating health disparities with	• Use language confidently (Skill #1a)<br><br>• Conduct self-management coaching (Skill #2b)<br><br>• Work as a team member (Skill #2d)<br><br>• Work in ways that increase individual and community empowerment (Skill #4b)	• Advocating for Individuals and Communities: Advocate for the needs and perspectives of communities (Role #5a)<br><br>• Build Individual and Community Capacity: Build individual capacity (Role #6a) Build community capacity (Role #6b) Training and building individual capacity with peers and among CHW groups (Role #6c)

	individual patients, family, community, and society<br>• Describe the role of CHWs in promoting social justice	• Use empowering and learner-centered teaching strategies (Skill #6a)<br>• Use a range of appropriate and effective educational techniques (Skill #6b)<br>• Facilitate group discussions and decision-making (Skill #6c)<br>• Seek out appropriate information and respond to questions about pertinent topics (Skill #6e)<br>• Find and share requested information (Skill #6f)<br>• Build and maintain a current resource inventory (Skill #8c)<br>• Apply critical thinking techniques and problem solving (Skill #9c)<br>• Use pertinent technology (Skill #9d)<br>• Pursue continuing education and life-long learning opportunities (Skill #9e)<br>• Participate in professional development of peer CHWs and in networking among CHW groups (Skill #9i)<br>• Understand social determinants of health and related disparities (Skill #11a)<br>• Understand pertinent health issues (Skill #11b)<br>• Knowledge about health behaviors theories (Skill #11e)	
Module # 9: **Engaging with Families & Communities**<br>Session 1:<br>**Working with Families**	• Discuss the diverse range of families that CHWs are likely to encounter<br>• Discuss frameworks Family-Community Health Promotion, Familismo, and Family Informed Trauma Treatment that analyzes the influence of family<br>• Discuss family engagement and how CHWs may stay actively engaged with patient's family members	• Use language confidently (Skill #1a)<br>• Conduct self-management coaching (Skill #2b)<br>• Work as a team member (Skill #2d)<br>• Help others identify goals and develop to their fullest potential (Skill #4a)<br>• Work in ways that increase individual and community empowerment (Skill #4b)	• Providing Coaching and Social Support: Motivating and encouraging people to obtain care and other services (Role #4b)<br>• Advocating for Individuals and Communities: Connecting to resources and advocating for basic needs (e.g. food and housing) (Role #5b)<br>• Building Individual and Community Capacity: Building individual capacity (Role #6a) Training and building

	• Identify best practices of family engagement and cultural perspectives of family<br>• Describe how CHWs keep a balance within the family structure<br>• Define home visiting and provide examples of when and why they are conducted<br>• Explain key concepts of family engagement through discussion of a case study	• Teach self-advocacy skills (Skill #4d)<br>• Speak up for individuals and communities (Skill #5c)<br>• Facilitate group discussions and decision-making (Skill #6c)<br>• Seek out appropriate information and respond to questions about pertinent topics (Skill #6e)<br>• Find and share requested information (Skill #6f)<br>• Participate in individual assessment through observation and active inquiry (Skill #7a)<br>• Apply critical thinking techniques and problem solving (Skill #9c)<br>• Use pertinent technology (Skill #9d)<br>• Pursue continuing education and life-long learning opportunities (Skill #9e)<br>• Maximize personal safety while working in community and/or clinical settings (Skill #9f)<br>• Participate in professional development of peer CHWs and in networking among CHW groups (Skill #9i)	individual capacity with peers and among CHW groups (Role #6c)<br>• <u>Implementing Individual and Community Assessments</u>: Participating in design, implementation, and interpretation of individual-level assessments (e.g. home environmental assessment) (Role #8a)<br>• <u>Outreach</u>: Follow-up on health and social service encounters with individuals, families, and community groups (Role #9b) Home visiting to provide education assessment, and social support (Role #9c)
Module # 9: **Engaging with Families & Communities**<br><br><u>Session 2:</u><br><br>**Working with Communities**	• Describe the terms community, community engagement, community organizing, and community advocacy<br>• Discuss the CHW's roles and responsibilities in the community capacity building<br>• Describe types of community partnerships and the role of CHWs within these partnerships<br>• Describe community engagement models Community Based Participatory Research, Participatory Action Research, and Community Action Model (CAM)	• Use language confidently (Skill #1a)<br>• Conduct self-management coaching (Skill #2b)<br>• Work as a team member (Skill #2d)<br>• Work in ways that increase individual and community empowerment (Skill #4b)<br>• Network, build community connections, and build coalitions (Skill #4c)<br>• Conduct community organizing (Skill #4e)<br>• Contribute to policy development (Skill #5a)<br>• Advocate for policy change (Skill #5b)	• <u>Cultural Mediation Among Individuals, Communities, and Health and Social Service Systems</u>: Educating individuals and communities about how to use health and social service systems (including understanding health and social services operate) (Role #1a)<br>• <u>Care Coordination, Case Management, and System Navigation:</u> Informing people and systems about community assets and challenges (Role #3e)<br>• <u>Advocating for Individuals and Communities</u>: Advocating for the needs and perspectives of communities (Role

		• Use empowering and learner-centered teaching strategies (Skill #6a)<br>• Use a range of appropriate and effective educational techniques (Skill #6b)<br>• Facilitate group discussions and decision-making (Skill #6c)<br>• Seek out appropriate information and respond to questions about pertinent topics (Skill #6e)<br>• Find and share requested information (Skill #6f)<br>• Participate in community assessment through observation and active inquiry (Skill #7b)<br>• Build and maintain a current resource inventory (Skill #8c)<br>• Apply critical thinking techniques and problem solving (Skill #9c)<br>• Use pertinent technology (Skill #9d)<br>• Pursue continuing education and life-long learning opportunities (Skill #9e)<br>• Maximize personal safety while working in community and/or clinical settings (Skill #9f)<br>• Participate in professional development of peer CHWs and in networking among CHW groups (Skill #9i)<br>• Apply the evidence-based practices of Community Based Participatory Research (CBPR) and Participatory Action Research (PAR) (Skill #10b)<br>• Knowledge about the community served (Skill #11g)	#5a) Conducting policy advocacy (Role #5c)<br>• <u>Building Individual and Community Capacity</u>: Building community capacity (Role #6b) Training and building individual capacity with peers and among CHW groups (Role #6c)<br>• <u>Implementing Individual and Community Assessments</u>: Participating in design, implementation, and interpretation of community-level assessments (e.g. windshield survey of community assets and challenges and community asset mapping) (Role #8b)<br>• <u>Participating in Evaluation and Research</u>: Identifying and engaging community members as research partners, including community consent processes (Role #10b)
Module # 10: **The Humanistic Value of Care Management**<br><u>Session 1:</u><br>**Humanistic Values in Health Care**	• Define humanity and discuss examples of humanity in health care settings<br>• Describe the patient's experience in navigating the health care system<br>• Discuss the history of mistrust from the patient's perspective in	• Use language confidently (Skill #1a)<br>• Conduct self-management coaching (Skill #2b)<br>• Work as a team member (Skill #2d)<br>• Coordinate care (including identifying and accessing resources and overcoming barriers) (Skill #3a)	• <u>Providing Coaching and Social Support</u>: Motivating and encouraging people to obtain care and other services (Role #4b)<br>• <u>Advocating for Individuals and Communities</u>: Advocating for the needs and perspectives of communities (Role

	the health care system and medical research<br>• Explain the role of the CHWs in providing care with a humanistic approach<br>• Read and discuss Buchanan's article on common good and influences on health behaviors	• Work in ways that increase individual and community empowerment (Skill #4b)<br>• Network, build community connections, and build coalitions (Skill #4c)<br>• Speak up for individuals and communities (Skill #5c)<br>• Facilitate group discussions and decision-making (Skill #6c)<br>• Seek out appropriate information and respond to questions about pertinent topics (Skill #6e)<br>• Find and share requested information (Skill #6f)<br>• Apply critical thinking techniques and problem solving (Skill #9c)<br>• Use pertinent technology (Skill #9d)<br>• Pursue continuing education and life-long learning opportunities (Skill #9e)<br>• Participate in professional development of peer CHWs and in networking among CHW groups (Skill #9i)	#5a) Connecting to resources and advocating for basic needs (e.g. food and housing) (Role #5b)<br>• <u>Building Individual and Community Capacity</u>: Building individual capacity (Role #6a) Training Building community capacity (Role #6b) Training and building individual capacity with peers and among CHW groups (Role #6c)<br>• <u>Outreach</u>: Follow-up on health and social service encounters with individuals, families, and community groups (Role #9b)
Module # 10: **The Humanistic Value of Care Management**<br><u>Session 2:</u><br>**CHW's Role of Care Coordination**	• Define care coordination for CHWs<br>• Describe examples of how CHWs engage with patients and collaborate with the healthcare team<br>• Discuss the CHW care coordination toolkit of (1) understanding patients' social determinants of health, (2) identifying how to manage care using empowerment approaches, (3) reflecting on CHWs role in care management<br>• Identify examples of compassionate care in the healthcare setting	• Use language confidently (Skill #1a)<br>• Conduct self-management coaching (Skill #2b)<br>• Work as a team member (Skill #2d)<br>• Coordinate care (including identifying and accessing resources and overcoming barriers) (Skill #3a)<br>• Make appropriate referrals (Skill #3b)<br>• Facilitate development of an individual and/or group action plan and goal attainment (Skill #3c)<br>• Coordinate CHW activities with clinical and other community services (Skill #3d)<br>• Follow-up and track care and referral outcomes (Skill #3e)	• <u>Care Coordination, Case Management, and System Navigation</u>: Participating in care coordination and/or case management (Role #3a) Making referrals and providing follow-up (Role #3b) Facilitating transportation to services and helping address barriers to services (Role #3c) Documenting and tracking individual and population level data (Role #3d) Informing people and systems about community assets and challenges (Role #3e)<br>• <u>Providing Coaching and Social Support</u>: Motivating and encouraging people to obtain care and other services (Role #4b) Supporting self-management of disease prevention and management

		• Use empowering and learner-centered teaching strategies (Skill #6a)<br>• Use a range of appropriate and effective educational techniques (Skill #6b)<br>• Facilitate group discussions and decision-making (Skill #6c)<br>• Seek out appropriate information and respond to questions about pertinent topics (Skill #6e)<br>• Find and share requested information (Skill #6f)<br>• Apply critical thinking techniques and problem solving (Skill #9c)<br>• Use pertinent technology (Skill #9d)<br>• Pursue continuing education and life-long learning opportunities (Skill #9e)<br>• Participate in professional development of peer CHWs and in networking among CHW groups (Skill #9i)	of health conditions (including chronic disease) (Role #4c)<br>• Building Individual and Community Capacity: Building individual capacity (Role #6a) Training Building community capacity (Role #6b) Training and building individual capacity with peers and among CHW groups (Role #6c)
